# Construction of a Yeast Two-Hybrid cDNA Library from Soybean Seeds at Different Developmental Stages and Screening of *SW16.1* Interacting Proteins

**DOI:** 10.3390/plants15213414

**Published:** 2026-11-03

**Authors:** Xingchen Liu, Mingzhu Zhao, Yiyang Li, Qingyuan He, Xue Yang, Dagang Wang, Yanlong Liu, Chenxi Fei, Xianlian Chen, Yingjie Shu

**Affiliations:** 1 College of Agriculture, Anhui Science and Technology University, Chuzhou 233100, China; 2 College of Biomedical and Health Sciences, Anhui Science and Technology University, Chuzhou 233100, China; 3 Crop Research, Anhui Academy of Agricultural Sciences, Hefei 230001, China

**Keywords:** *Glycine max*, seed weight, yeast two-hybrid cDNA library, *GmSW16.1*, protein–protein interaction

## Abstract

Seed weight represents a critical yield-associated quantitative trait in soybean (*Glycine max*) and is governed by polygenic regulatory networks. Although several seed-weight-related genes have been functionally characterized, the interactome of the major seed-weight determining gene *SW16.1* remains largely unexplored. In this study, we constructed a yeast two-hybrid (Y2H) cDNA library using pooled total RNA isolated from developing seeds of the soybean cultivar Nannong 1138-2 covering five key seed-development stages. Using GmSW16.1 (cultivated Nannong 1138-2 allelic) as bait, Y2H screening was performed. Following multi-round screening, Sanger sequencing, and filtering based on functional annotation, 11 candidate interactors assigned to nine distinct functional categories were recovered. Interactions were further verified through point-to-point Y2H assays, quantitative α-galactosidase (α-Gal) activity measurements, and bimolecular fluorescence complementation (BiFC). We confirmed that metallothionein-like protein 4A-related Glyma.18G157800 (GmMT4) and the papain-like cysteine protease Glyma.08G116300 physically interact with both GmSW16.1 and GsSW16.1 (wild soybean N24852). A two-glutamine polymorphism within the PolyQ (polyglutamine) repeat tract of these two allelic proteins did not abolish protein–protein interactions, yet significantly altered their interaction strength toward the above-mentioned partners. *Glyma.08G116300* displayed preferential transcript accumulation during soybean seed development, and its transcript abundance differed dynamically between the chromosome segment substitution line CSSL3068 and its cultivated parent Nannong 1138-2 across developmental stages. Heterologous overexpression of this gene in *Arabidopsis thaliana* significantly increased the 1000-seed weight (by 12–15%) and increased seed length and width. This study delivers a seed-development-specific Y2H library resource for soybean research. We put forward a testable working hypothesis describing a dual-layer regulatory module integrating transcriptional control and direct protein–protein interaction for the GmSW16.1-GmMT4 regulatory node. These findings advance our mechanistic understanding of how natural PolyQ allelic variation shapes soybean seed-weight performance and provides candidate gene materials for soybean molecular design breeding.

## 1. Introduction

Soybean (*Glycine max* (L.) Merr.) is one of the globally dominant oil and protein producing crops. Its seeds supply more than half of worldwide oilseed output and contribute over one quarter of dietary and livestock feed protein resources [1]. Seed weight is a core agronomic trait determining soybean yield and market quality and was subjected to strong artificial selection throughout soybean domestication. During domestication, the seed weight of cultivated soybean increased from approximately 2 g in wild soybean up to 16–20 g in modern cultivars [2,3,4]. Nevertheless, seed weight is a complex quantitative trait controlled by multiple genes and is highly susceptible to environmental fluctuations. Numerous quantitative trait loci (QTLs) linked to seed-weight variation have been mapped across the soybean genome; however, only a small subset of the underlying genes have been cloned and experimentally validated [5]. This bottleneck restricts mechanistic dissection and precision genetic improvement for seed-weight traits. Accordingly, identifying key genes and their regulatory networks controlling seed weight carries both theoretical significance and practical breeding value.

Multiple genes modulating seed size and seed weight in soybean have been isolated via map-based cloning and genome-wide association studies. The sugar transporter encoding genes *GmSWEET10a/b* are domestication targets and coordinately modulate seed dimension, oil accumulation, and protein content [6]. The deubiquitinase gene *GmSW17* regulates seed size through histone H2B monoubiquitination [7]. The NF-YA transcription factor *SW14* fine-tunes seed weight and quality by repressing *GmLEC1*-dependent transcriptional activation [8]. The protein phosphatase gene *PP2C-1* positively elevates seed weight by promoting seed-coat cell expansion [9]. The basic leucine zipper (bZIP) transcription factor *GmSW19* directly suppresses transcription of the positive seed-weight regulator *GmSW5*, whereas *GmSK21* enhances GmSW19 phosphorylation and protein stability to reduce seed weight [10]. Additional seed-weight-related regulators include *GmCYP78A72*, *ST1*, *GmST05*, and *SoyWRKY15a* [11,12,13,14]. Cumulatively, these studies demonstrate that soybean seed-weight regulation relies on sophisticated multilayered networks integrating transcriptional reprogramming, phytohormone signaling, sugar metabolism, and protein homeostasis [5,15,16]. Investigating individual genetic pathways alone cannot fully unravel the regulatory landscape governing seed size; hence, complementary insights from the perspective of protein–protein interactomics are urgently needed.

The major effect seed-weight gene *SW16.1* was previously identified through map-based cloning and encodes a LIM-domain-containing protein. At least 12 allelic variants of *SW16.1* exist in natural soybean germplasm. Polymorphisms altering glutamine repeat numbers within the PolyQ tract substantially modify transcript abundance of the metallothionein-coding gene *GmMT4* in developing seeds [17]. Both the cultivated soybean allele *GmSW16.1* (Nannong 1138-2) and wild soybean allele *GsSW16.1* (accession N24852) bind the ABA-responsive element (ABRE) cis-element located in the *GmMT4* promoter and trigger its transcriptional activation. The loss of deleterious *SW16.1* haplotypes during domestication represents a critical genetic driver for seed-weight enlargement in cultivated soybean [17]. Plant LIM-domain proteins participate in diverse plant developmental programs [18]. Length variations in low-complexity PolyQ repeats can reshape protein helical conformation, thereby modifying transcriptional activity and protein binding capacities [19]. PolyQ-rich low-complexity domains may also confer prion-like biochemical properties and alter cellular protein functional states [20]. Collectively, these observations raise the hypothesis that allelic variation at *SW16.1* may exert effects not merely by adjusting downstream gene transcription but also by remodeling protein–protein interaction repertoires. To date, functional studies of *SW16.1* have mainly focused on its function as a transcriptional regulator, and its protein-level interactome remains largely uncharacterized.

Yeast two-hybrid screening represents a well-established large-scale experimental platform for identifying protein–protein interactions and is widely deployed in plant interactomic research [21]. Several tissue-specific Y2H cDNA libraries have been constructed for soybean [22], including libraries derived from developing seeds [23]. Even so, no dedicated seed-development-focused Y2H library has been generated for screening interactors of seed-weight-related genes such as *GmSW16.1*. Soybean seed development is accompanied by profound spatiotemporal transcriptomic reprogramming [24]. Pooling equal-quantity RNA isolated from successive seed-development stages maximizes recovery of dynamically expressed transcripts and mitigates the risk of missing stage-specific transcripts that would occur using single-time-point sampling.

Given the above background, this study was designed to dissect the protein interaction network of *GmSW16.1* and test whether PolyQ allelic variation modulates protein binding properties during soybean seed development. We constructed a high-quality Y2H cDNA library using RNA pooled from five representative seed-development stages of the soybean cultivar Nannong 1138-2. After rigorous bait quality assessment for autoactivation and toxicity, library screening was performed using BD-GmSW16.1 as bait. Candidate interactors were filtered, sequenced, and subjected to multi-system interaction validation. For the high-priority candidate *Glyma.08G116300*, bioinformatic characterization, expression profiling, and heterologous functional assays in *Arabidopsis* were conducted. The objectives of this work were: (i) to generate a reusable interactomic resource for soybean seed-development research; (ii) to identify bona fide protein interactors of GmSW16.1; (iii) to test the working hypothesis of a dual-layer regulatory module combining transcriptional and protein interaction-mediated control for the *GmSW16.1*-*GmMT4* node; and (iv) to uncover candidate gene resources supporting soybean seed-weight-improvement breeding.

## 2. Results

### 2.1. Construction and Quality Evaluation of the Seed-Development Y2H cDNA Library

**Figure 1:**
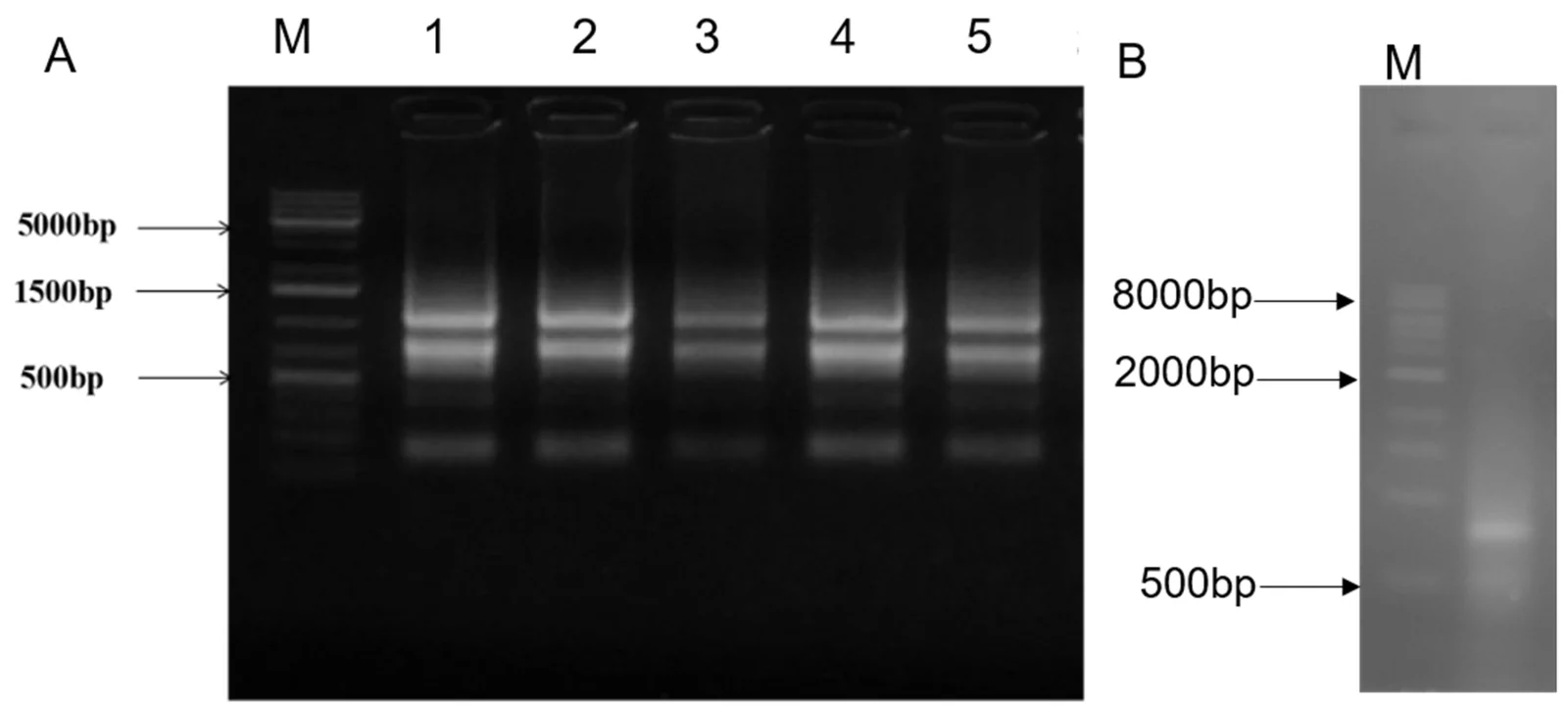
Total RNA extraction and double-stranded cDNA detection from developing soybean seeds. (**A**) Total RNA isolated from Nannong 1138-2 seeds collected at 14, 21, 28, 35, and 42 DAF (lanes 1–5). M: 10 kb DNA ladder marker. The three prominent bands from top to bottom correspond to 28S, 18S, and 5S rRNA. (**B**) Double-stranded cDNA amplified by LD-PCR, showing a size distribution from approximately 300 to 6000 bp. M: DNA size marker.

To construct a representative Y2H cDNA library for soybean seed development, total RNA was extracted from Nannong 1138-2 seeds harvested at five successive developmental stages (14, 21, 28, 35, and 42 DAF). Agarose gel electrophoresis resolved three distinct rRNA bands corresponding to 28S, 18S, and 5S rRNA, with no visible degradation products in any of the five samples (Figure 1A). Spectrophotometric quantification yielded RNA concentrations ranging from 289.14 to 414.52 ng/μL (Table 1). All samples exhibited A260/A280 and A260/A230 ratios above 2.0 (Table 1), indicating minimal contamination by proteins, phenolic compounds, or salts. Equal-mass RNA aliquots from each developmental stage were pooled to a total input of 1.2 μg.

Double-stranded cDNA was amplified by SMART-based LD-PCR and resolved on an agarose gel, which produced a broad smear spanning approximately 300 to 6000 bp (Figure 1B), indicating a wide distribution of cDNA insert sizes. The purified ds-cDNA was co-transformed with linearized pGADT7-Rec into competent Y187 yeast cells. The resulting library titer was 2 × 10^7^ cfu/mL, exceeding the 1 × 10^7^ cfu/mL benchmark for high-quality Y2H libraries. These quality metrics indicate that the library possesses sufficient capacity and insert-size diversity for downstream interaction screening.

### 2.2. Autoactivation and Toxicity Assessment of BD-GmSW16.1 and BD-GsSW16.1 Bait Constructs

**Figure 2:**
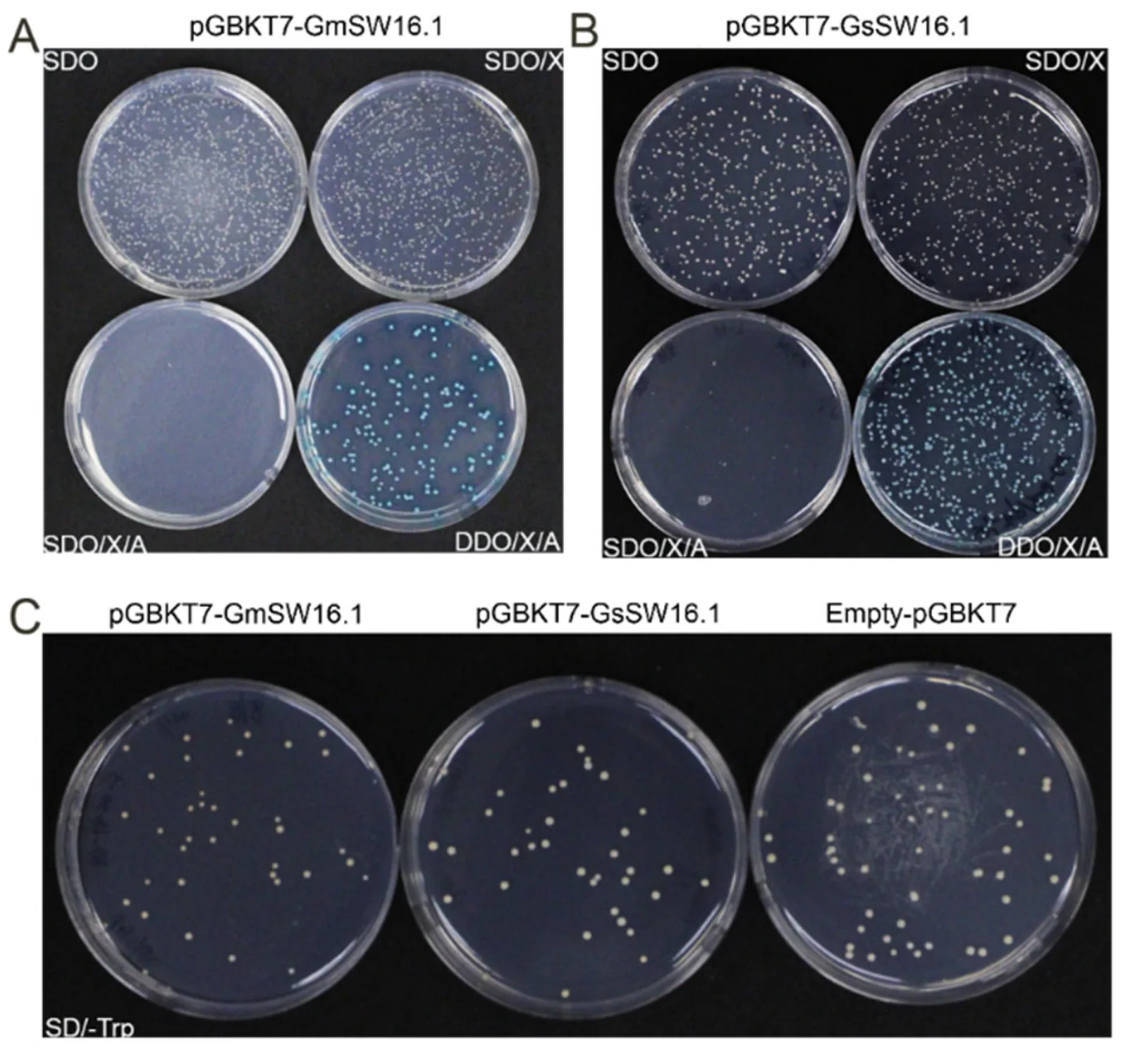
Autoactivation and toxicity assays for bait constructs BD-GmSW16.1 and BD-GsSW16.1. (**A**,**B**) Autoactivation tests on selective agar plates. Bait strains were diluted to 10^−1^; the positive-control combination pGBKT7-53 + pGADT7-T was diluted to 10^−3^. (**C**) Toxicity growth assay on SD/−Trp medium. Empty pGBKT7 served as the negative control (10^−3^ dilution). SDO is SD/−Trp plate, SDO/X is SD/−Trp/X-α-Gal plate, SDO/X/A is SD/−Trp/X-α-Gal/AbA plate; DDO/X/A is SD/−Trp/-Leu/X-α-Gal/AbA. Three independent biological replicates yielded consistent results.

Before library screening, the bait constructs BD-GmSW16.1 and BD-GsSW16.1 were tested for autoactivation and toxicity in Y2HGold yeast cells. On the SDO and SDO/X plates, yeast strains harboring either bait construct produced abundant white colonies, indicating that the reporter genes were not activated. On the SDO/X/A medium containing aureobasidin A, no colonies were observed for either bait construct. In contrast, the positive-control combination (pGBKT7-53 + pGADT7-T) produced blue colonies on DDO/X/A plates (Figure 2A,B). These results demonstrate that neither BD-GmSW16.1 nor BD-GsSW16.1 exhibits autoactivating capacity.

For toxicity assessment, yeast strains expressing BD-GmSW16.1 or BD-GsSW16.1 were plated on SD/−Trp medium alongside a strain transformed with empty pGBKT7. Colony size and proliferation rate were comparable across all three strains (Figure 2C), indicating that neither bait fusion protein is toxic to yeast cells. Both bait constructs therefore meet the quality criteria required for large-scale library screening.

### 2.3. Y2H Library Screening and Identification of Candidate Interacting Genes

**Figure 3:**
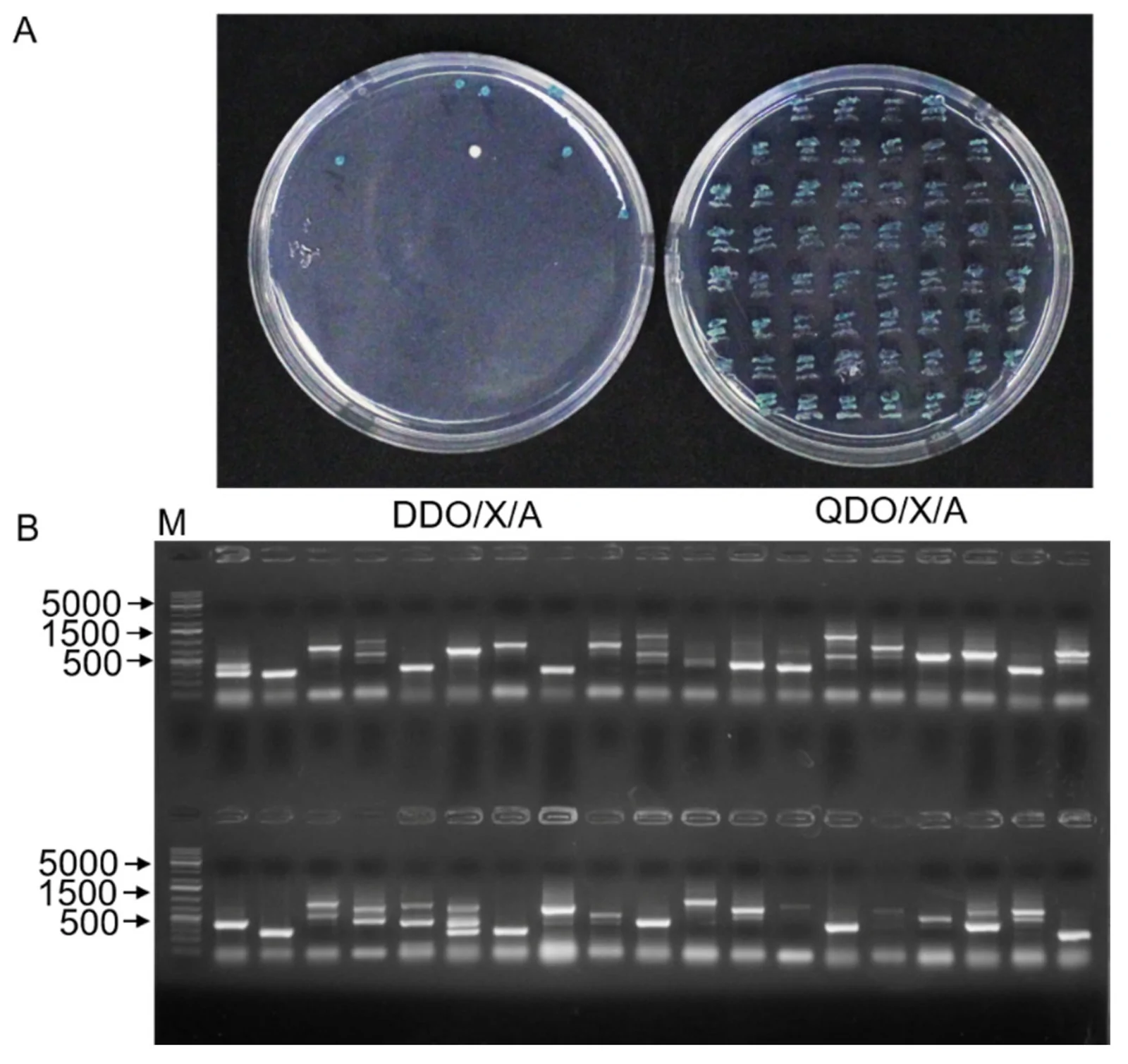
Yeast two-hybrid library screening workflow and prey insert colony PCR detection. (**A**) Representative plate photographs of primary low-stringency screening (DDO/X/A) and secondary high-stringency screening QDO/X/A (SD/−Trp/−Leu/−His/−Ade + X-α-Gal + AbA). (**B**) Colony PCR amplification of prey cDNA inserts recovered from positive yeast clones. M: DNA size marker.

Large-scale library screening was performed using BD-GmSW16.1 as bait. Yeast mating between the bait-carrying Y2HGold strain and the library-containing Y187 strain yielded a mating efficiency of 2.4% (number of diploid colonies on DDO plates/total input haploid bait and library cell count × 100%), meeting the standard for Y2H library screening. Approximately 200 blue colonies were recovered on low-stringency DDO/X/A plates as primary positives. All primary positives were re-streaked onto high-stringency QDO/X/A plates for secondary screening, yielding 110 surviving positive clones (secondary-screen retention rate approximately 55%; Figure 3A).

Colony PCR targeting prey-vector inserts was performed for all 110 secondary-screen survivors (Figure 3B). Clones with identical insert fragment sizes were preliminarily treated as redundant and discarded. Seventy non-redundant yeast clones were selected for plasmid isolation and Sanger sequencing. Insert sequences were annotated by BLAST searches against the soybean reference genome assembly. Eighteen clones without valid genome matches and fourteen clones carrying erroneous ORFs were eliminated. Thirty-eight clones retained correctly framed prey inserts; after removal of 27 redundant sequences, 11 unique candidate interacting genes were obtained (Table 2).

Functional annotation assigned these 11 candidates to nine distinct functional categories: alcohol dehydrogenase-related proteins, Bowman–Birk serine protease inhibitor family members, S-adenosylmethionine synthase, ubiquinol–cytochrome c reductase subunits, nucleotide diphosphatase/pyrophosphatase, SF2-CUPIN-domain-containing proteins, papain-like cysteine proteases, plant PEC-type metallothioneins, and one gene with unknown biological function (Table 2). Candidate prioritization for downstream validation was based on two criteria: (1) functional annotation of the gene, and (2) documented biological roles of the corresponding *Arabidopsis* homologs in seed development or metabolic processes [5,16,25]. Genes with functionally uncharacterized *Arabidopsis* homologs were not subjected to further validation. Following this filtering workflow, five high-priority candidates were selected, Glyma.14G156400, Glyma.16G062100, Glyma.05G222200, Glyma.08G116300, and Glyma.18G157800, which were advanced to point-to-point interaction assays.

### 2.4. Validation of Physical Interactions Between GmSW16.1/GsSW16.1 and Candidate Partners

**Figure 4:**
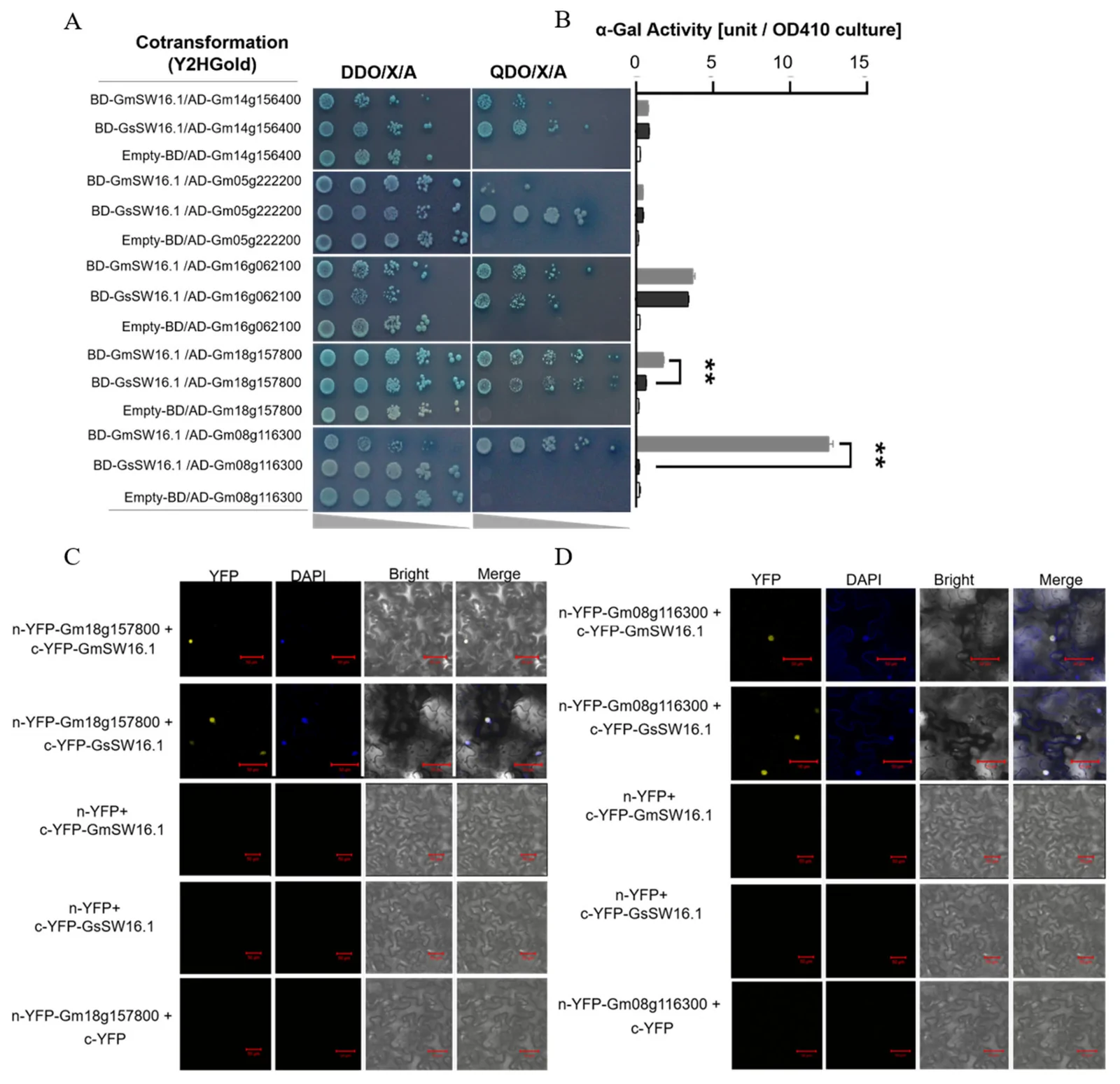
Multi-platform validation of protein–protein interactions. (**A**) Point-to-point Y2H interaction assays. Five AD-fused candidate proteins were co-transformed with BD-GmSW16.1 or BD-GsSW16.1 and grown on DDO/X/A and QDO/X/A plates; empty-BD served as negative control. Gray trapezoid marks 10-fold serial dilutions (10^−1^ to 10^−5^). (**B**) Quantitative α-galactosidase reporter activity measurements. Bars represent mean ± SD from three independent biological replicates. Asterisks denote statistically significant differences by one-way ANOVA with Tukey’s post hoc test: ** *p* < 0.01. (**C**,**D**) BiFC interaction validation for GmSW16.1/GsSW16.1 paired with GmMT4 and Glyma.08G116300, respectively. YFP fluorescence was imaged 24 h post-agroinfiltration; cell nuclei were stained with DAPI. Negative-control combinations are nYFP + cYFP-GmSW16.1, nYFP + cYFP-GsSW16.1, nYFP-GmMT4 + empty-cYFP and nYFP-Glyma.08G116300 + empty-cYFP. Scale bars = 50 μm.

Full-length CDSs of the five prioritized candidate genes were individually cloned into the pGADT7-AD prey vector. Each AD-candidate construct was co-transformed separately with BD-GmSW16.1, BD-GsSW16.1, or empty-BD negative control into Y2HGold competent yeast cells. Serially diluted yeast suspensions (10^−1^ to 10^−5^) were spotted onto DDO/X/A and QDO/X/A selective agar plates (Figure 4A). Blue colonies were observed for all five candidates when co-expressed with either BD-GmSW16.1 or BD-GsSW16.1, whereas no blue colonies appeared in empty-BD negative-control combinations. These point-to-point Y2H results indicate that the two-glutamine PolyQ allelic difference does not abolish the occurrence of physical interaction between SW16.1 and these candidate partners in a heterologous yeast system.

To determine whether PolyQ-region allelic variation modulates interaction strength rather than interaction existence, quantitative α-galactosidase (α-Gal) reporter activity measurements were performed (Figure 4B). Significant differences in reporter activity between BD-GmSW16.1 and BD-GsSW16.1 co-transformations were detected exclusively for two candidates: Glyma.18G157800 (GmMT4) and Glyma.08G116300. For GmMT4, co-expression with BD-GmSW16.1 yielded significantly higher α-Gal activity than co-expression with BD-GsSW16.1. For Glyma.08G116300, the difference was more pronounced. The remaining three candidates exhibited no statistically significant shifts in interaction strength between the two allelic bait proteins.

Since only these two candidates displayed PolyQ-dependent quantitative changes in binding strength, GmMT4 and Glyma.08G116300 were selected for further in planta BiFC validation in *Nicotiana benthamiana* leaf epidermal cells. When nYFP-GmMT4 and nYFP-Glyma.08G116300 were each co-expressed with cYFP-GmSW16.1 or cYFP-GsSW16.1, distinct yellow fluorescent protein (YFP) signals were detected inside nuclei and co-localized with DAPI nuclear-staining signals (Figure 4C,D). By contrast, no YFP fluorescence was detected for any negative-control combinations (nYFP + cYFP-GmSW16.1; nYFP + cYFP-GsSW16.1; nYFP-GmMT4 + empty-cYFP; nYFP-Glyma.08G116300 + empty-cYFP). These BiFC observations independently confirm physical interactions between GmSW16.1/GsSW16.1 and GmMT4/Glyma.08G116300 within living plant cells.

### 2.5. Molecular Characterization and Heterologous Functional Assays for Glyma.08G116300

**Figure 5:**
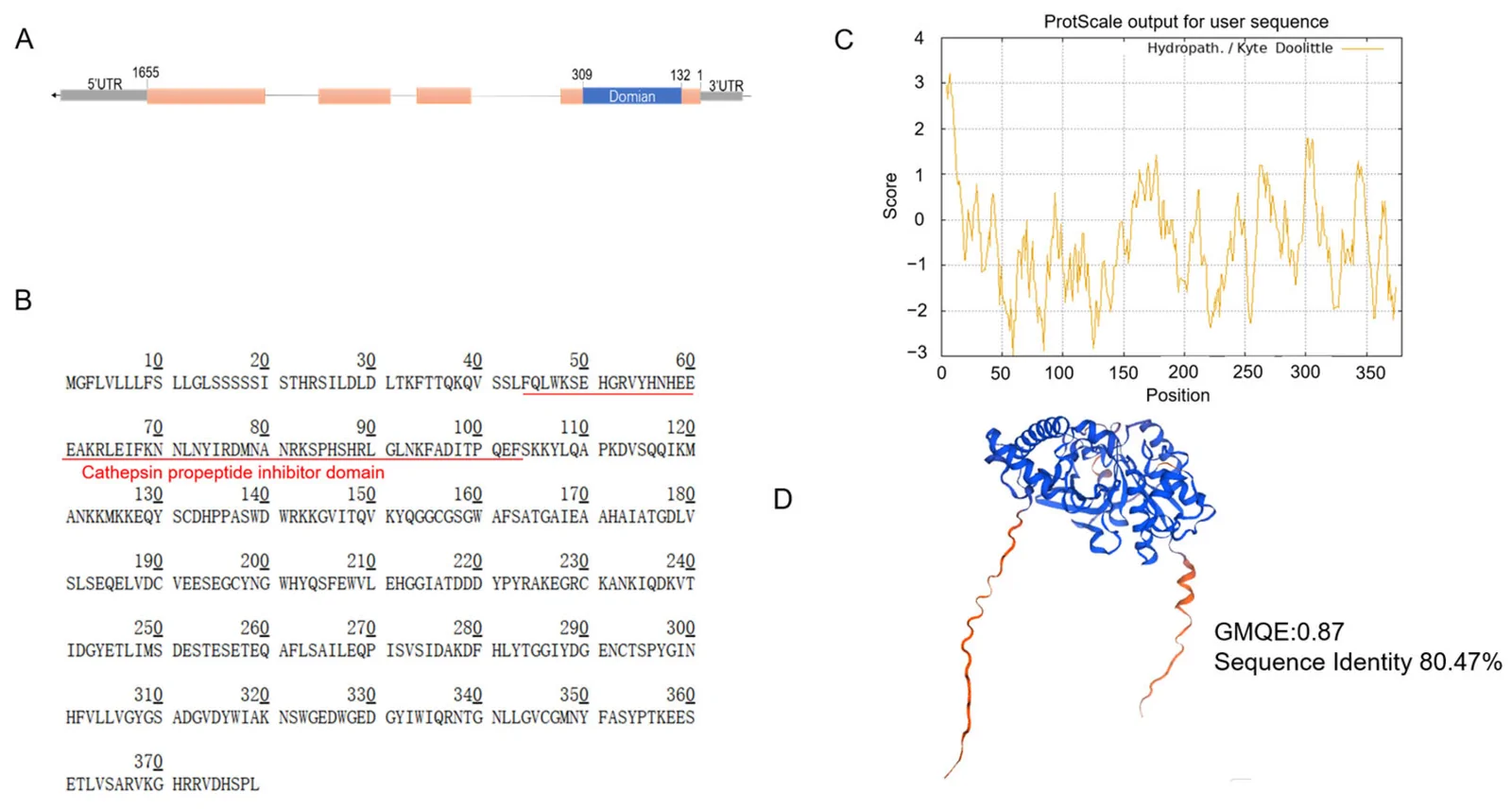
Molecular feature prediction for Glyma.08G116300. (**A**) Gene structure schematic for Glyma.08G116300; blue box marks the functional domain; numbers above boxes denote amino acid positions. (**B**) Deduced full-length amino acid sequence; red underline highlights the cathepsin propeptide-inhibitor domain. (**C**) Kyte Doolittle hydropathy profile. (**D**) Homology-derived three-dimensional protein-structure prediction. GMQE value is 0.84; sequence identity with template is 80.47%.

Previous studies have documented that transcript levels of *GmMT4* (*Glyma.18G157800*) correlate positively with seed weight in soybean [17]. We next focused molecular and functional characterization on the second high-confidence interactor, *Glyma.08G116300*. Glyma.08G116300 encodes a papain-like cysteine protease belonging to the PLCP (papain-like cysteine protease) superfamily and harbors a cathepsin propeptide-inhibitor domain. The predicted protein product consists of 379 amino-acid residues (Figure 5A,B). ProtParam physicochemical prediction indicated an instability index of 41.68, classifying this protein as unstable. Its aliphatic index was 74.12, suggesting moderate thermal lability, and the grand average of hydropathicity (GRAVY) was −0.524, indicative of an overall hydrophilic character (Figure 5C). The SWISS-MODEL-generated homology model exhibited a global model quality estimate (GMQE) of 0.84 and 80.47% sequence identity against the modeling template, supporting reasonable model reliability (Figure 5D).

**Figure 6:**
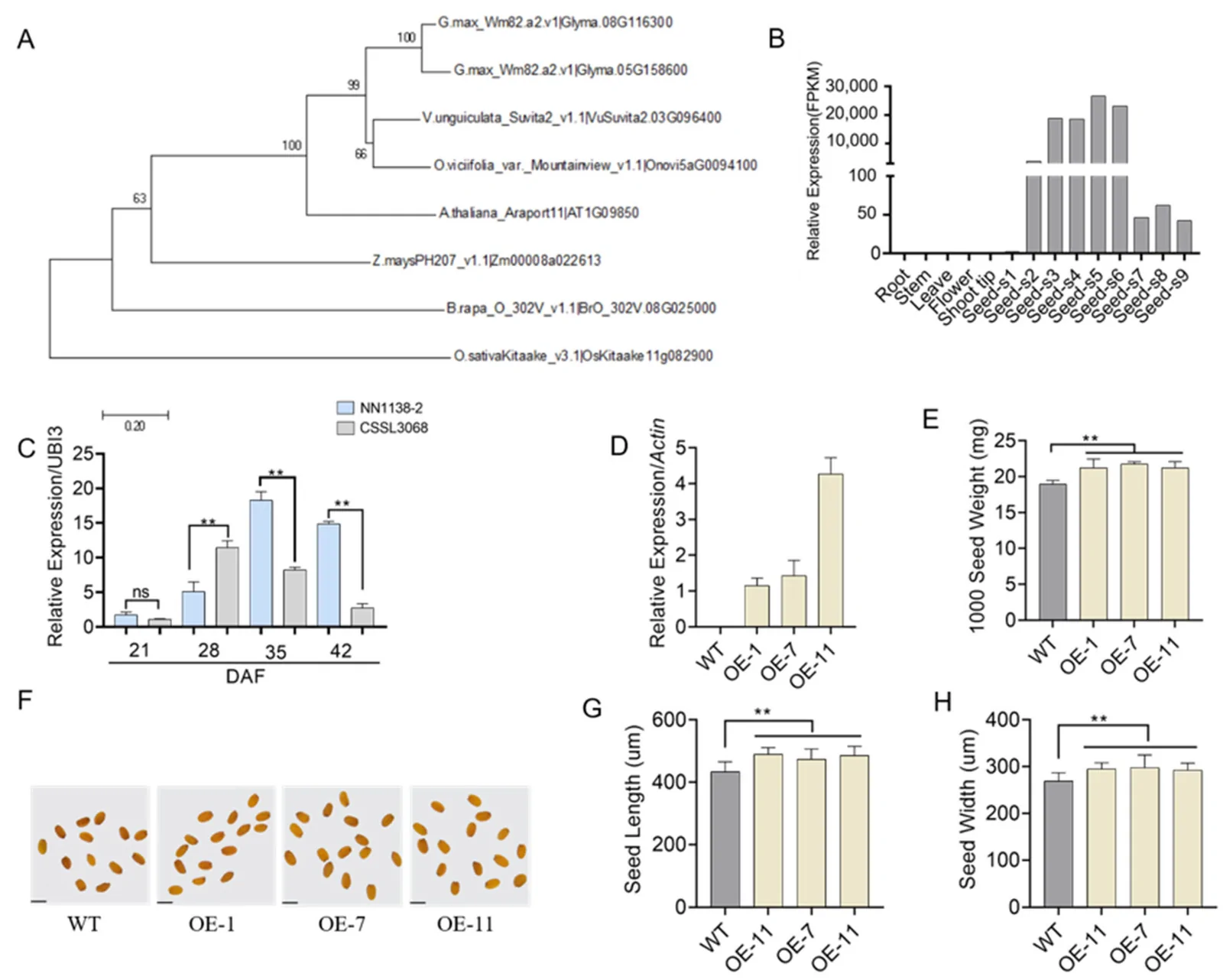
Phylogenetic analysis, expression profiling, and heterologous functional validation of *Glyma.08G116300*. (**A**) Neighbor-joining phylogenetic tree of *Glyma.08G116300* homologous proteins. (**B**) Tissue- and seed-stage-specific expression profile, fragments per kilobase of transcript per million mapped reads (FPKM) values retrieved from Phytozome. (**C**) Relative transcript abundance of *Glyma.08G116300* in the seeds of Nannong 1138-2 (NN1138-2) and CSSL3068 at different developmental stages. The soybean *UBI3* gene was used as the internal control. *n* = 3 biological replicates. (**D**) Relative transcript abundance of *Glyma.08G116300* in three independent *Arabidopsis* overexpression lines; *AtActin2* was used as internal reference; values represent mean ± SD for three biological replicates. (**E**) Statistical comparison of 1000-seed weight between overexpression lines and wild-type (WT) Col-0. *n* = 3 biological replicates per line, each based on 1000 individually counted seeds. (**F**) Representative photographs of the mature seeds of the WT, OE-1, OE-7 and OE-11. Scale bar, 1 cm. (**G**,**H**) Seed length and width of the WT, OE-1, OE-7 and OE-11. *n* = 15 independent replicates. Bars denote mean ± SD; ** *p* < 0.01 by one-way ANOVA with Tukey’s post hoc test. ns, not significant.

Phylogenetic reconstruction demonstrated that evolutionary divergence of this gene family largely coincides with the monocot dicot separation during plant evolution, and orthologs are highly conserved within legume species (Figure 6A). Public Phytozome expression datasets show that *Glyma.08G116300* transcripts accumulate predominantly during early-to-middle seed-development stages (Seed-s1 to Seed-s5), peaking at the Seed-s5 stage, while transcript abundance remains very low in vegetative tissues and floral organs (Figure 6B). We further analyzed the relative transcript abundance of *Glyma.08G116300* in the two parents of the chromosome segment substitution line at different seed developmental stages (Figure 6C). Transcript levels did not differ significantly between the two parents at the early stage (21 DAF). At the mid-stage (28 DAF), expression in CSSL3068 was significantly higher than that in the cultivated soybean Nannong 1138-2 (NN1138-2). By contrast, expression in NN1138-2 was significantly higher than in CSSL3068 at later stages (35 and 42 DAF). These results suggest that *Glyma.08G116300* may be involved in soybean seed development.

To assess its biological activity, we generated *Arabidopsis Col-0* transgenic plants overexpressing *Glyma.08G116300*. Three independent homozygous T_4_ overexpression lines (OE-1, OE-7 and OE-11) with elevated transgene expression were selected for phenotyping (Figure 6D). We observed that the 1000-seed weight of overexpression lines increased by 12–15% compared with the WT control (Figure 6E). The seed length and width of the transgenic lines were significantly greater than those of the WT (Figure 6F–H). These results indicate that *Glyma.08G116300* positively influences seed development in a heterologous background.

## 3. Discussion

### 3.1. A Seed-Development-Specific Y2H Library as a Reusable Interactomic Resource for Soybean Seed-Weight Research

Tissue- and developmental-stage-specific cDNA libraries coupled with yeast two-hybrid (Y2H) screening remain a powerful strategy for mapping protein–protein interaction networks in plants [21]. In this study, we generated a Y2H cDNA library from pooled RNA isolated from five successive seed-development stages of the soybean cultivar Nannong 1138-2. The library reached a titer of 2 × 10^7^ cfu/mL and contained inserts ranging from approximately 300 to 6000 bp, thereby meeting the standard quality criteria for large-scale Y2H screening. Compared with previously reported soybean Y2H libraries, which were often constructed from vegetative tissues or stress-treated materials [22], our library was purpose-built for seed ontogeny. This distinction is important because soybean seed development involves profound spatiotemporal transcriptomic reprogramming [24], and a library representing multiple developmental stages is better suited to capturing dynamically expressed transcripts than a single-stage library.

The multi-stage RNA pooling strategy offers a practical balance between representativeness and experimental tractability. By combining equal RNA amounts from 14, 21, 28, 35, and 42 DAF, the library increases the likelihood of recovering transcripts that are active at different phases of seed development, including early embryo patterning, middle maturation, and late desiccation-related processes. At the same time, pooling may dilute low-abundance transcripts that are restricted to a narrow developmental window. Future efforts could therefore complement this resource with stage-specific or single-cell libraries to capture rare or transiently expressed interactors. Nevertheless, the current library constitutes a reusable interactomics resource that can be screened not only with GmSW16.1 but also with other seed-weight-related proteins. This resource-oriented contribution is conceptually important because most soybean seed-weight studies have focused on genetic mapping, transcriptional regulation, or downstream phenotypic validation, whereas systematic protein-interaction resources for developing seeds remain limited.

### 3.2. The GmSW16.1 Interactome Suggests Multi Pathway Coordination in Soybean Seed-Weight Control

Our screening recovered 11 unique candidate GmSW16.1-interacting proteins that fall into nine functional categories, including alcohol dehydrogenase-related proteins, Bowman–Birk serine protease inhibitors, S-adenosylmethionine synthase, ubiquinol–cytochrome c reductase subunits, nucleotide diphosphatase/pyrophosphatase, SF2-CUPIN-domain proteins, papain-like cysteine proteases, plant PEC-type metallothioneins, and one uncharacterized protein. This functional diversity is consistent with the emerging view that soybean seed size and weight are governed by interconnected regulatory networks rather than linear genetic pathways [5,15,16]. Known seed-weight regulators include sugar transporters such as *GmSWEET10a/b* [6], the deubiquitinase *GmSW17* [7], the nuclear factor Y subunit A (NF-YA) transcription factor *SW14* [8], the protein phosphatase *PP2C-1* [9], the GmSW19–GmSW5–GmSK21 module [10], and additional regulators such as *GmCYP78A72*, *ST1*, *GmST05*, and *SoyWRKY15a* [11,12,13,14]. These proteins act through transcriptional control, hormone signaling, sugar metabolism, protein homeostasis, and seed-coat development. Our results add a complementary protein interaction layer to this landscape and suggest that *GmSW16.1* may function as a molecular hub that coordinates several of these processes through direct physical associations.

This interpretation is supported by the functional annotation of the candidate interactors. The recovery of GmMT4, a metallothionein involved in metal homeostasis and redox regulation [25,26,27], and *Glyma.08G116300*, as a Papain-like cysteine peptidase superfamily gene, points to possible links between GmSW16.1 and proteolytic regulation, metal-ion homeostasis, and cellular redox balance during seed development. The LIM domain of GmSW16.1 may provide a scaffold-like interface that enables it to associate with functionally distinct partners [18]. However, Y2H screening is performed in a heterologous yeast environment and is inherently prone to false-positive interactions. Therefore, only candidates supported by multiple orthogonal assays should be regarded as high-confidence interactors. In this study, GmMT4 and Glyma.08G116300 were validated by point-to-point Y2H, X-α-Gal and BiFC, whereas the remaining nine candidates await further validation. The prioritization strategy, which relied partly on the known functions of *Arabidopsis* homologs, may also have excluded soybean-specific or novel interactors without well-characterized reference homologs. Future studies should therefore combine unbiased interaction screening with domain-based, evolutionary, and tissue-specific prioritization to avoid overlooking non-canonical partners.

### 3.3. A Dual-Layer GmSW16.1–GmMT4 Regulatory Module Integrating Transcriptional and Protein-Level Control

Previous genetic and molecular work demonstrated that *GmSW16.1* acts as a transcriptional activator that directly binds the ABRE cis-element in the *GmMT4* promoter and promotes *GmMT4* transcription; *GmMT4* transcript abundance is positively correlated with soybean seed weight [17]. Our study extends this model by showing that GmSW16.1 and its wild soybean allelic variant GsSW16.1 physically interact with the GmMT4 protein. Taken together, these findings support a testable working hypothesis of a dual-layer regulatory module in which *GmSW16.1* controls *GmMT4* function at two interconnected levels: transcriptional activation of the *GmMT4* gene and direct protein–protein interaction with the GmMT4 protein product. This hypothesis is conceptually novel because it moves beyond the prevailing view of *SW16.1* as primarily a transcription factor and suggests that allelic variation at this locus may influence seed development through both gene regulatory and protein interaction mechanisms.

The biological significance of this proposed module is reinforced by the known functions of metallothioneins. Plant metallothioneins participate in metal detoxification, metal-ion homeostasis, and reactive oxygen species management [25,26,27], processes that are closely linked to seed development, storage reserve accumulation, and stress tolerance. If SW16.1 physically associates with GmMT4 in developing seeds, this interaction could affect GmMT4 protein stability, metal-binding capacity, subcellular localization, or feedback on transcriptional outputs. Such a mechanism would provide a plausible link between allelic variation at *SW16.1* and quantitative changes in seed weight. However, this remains a working hypothesis rather than a proven regulatory mechanism. Direct in planta evidence is still required, including co-immunoprecipitation from developing soybean seeds, split luciferase or FRET assays in soybean protoplasts, and biochemical tests of GmMT4 stability and metal-binding activity in the presence and absence of GmSW16.1 or GsSW16.1.

### 3.4. PolyQ Length Variation Quantitatively Tunes, Rather than Abolishes, Protein–Protein Interactions

A central finding of this study is that the two-glutamine polymorphism within the PolyQ repeat tract of GmSW16.1 and GsSW16.1 did not abolish interactions with GmMT4 or Glyma.08G116300, but it significantly altered interaction strength toward these two partners. This distinction between interaction occurrence and interaction strength is important. It suggests that natural allelic variation in low-complexity regions can act as a quantitative modifier of protein–protein interaction networks rather than as a simple switch that rewires the entire interactome. The remaining candidate interactors showed no statistically significant allele-dependent differences in interaction strength, indicating that the effect of PolyQ length variation is partner-specific rather than global. It should be noted that the α-galactosidase reporter activity measured here reflects the interaction-associated reporter signal under the specific yeast assay conditions, is used comparatively as a proxy for interaction strength between alleles, and is not equivalent to a directly determined biochemical binding affinity, which would require in vitro binding assays with purified proteins.

Structural studies have shown that PolyQ repeats can adopt helical conformations stabilized by unconventional hydrogen bonds between glutamine side chains and the polypeptide backbone, with helical propensity increasing as repeat length increases [19]. Low-complexity PolyQ domains may also exhibit prion-like biochemical properties and influence protein functional states [20]. Based on these observations, we hypothesize that the gain or loss of two-glutamine residues is insufficient to disrupt the overall folding or global interaction selectivity of SW16.1, but it may remodel the local conformation surrounding the PolyQ-containing region. Such local remodeling could alter the accessibility of specific binding interfaces and selectively adjust affinity for partners such as *GmMT4* and *Glyma.08G116300*. This model complements previous genetic evidence that *SW16.1* allelic variation affects *GmMT4* transcription [17] and provides a plausible mechanistic explanation for how small coding-sequence polymorphisms can generate quantitative phenotypic divergence during soybean domestication. Nevertheless, this conformational interpretation is inferred from published structural studies and requires direct experimental validation using recombinant GmSW16.1 and GsSW16.1 proteins, including sequence variations that might amplify phenotypic effects through both ‘transcription level’ and ‘protein interaction strength’.

### 3.5. Glyma.08G116300 as a PLCP-Family Candidate Positively Influencing Seed Weight

Papain-like cysteine proteases constitute a large protease superfamily in plants and participate in diverse physiological processes, including storage protein remobilization during germination, programmed cell death, organ senescence, immune responses, and developmental reprogramming [28,29,30]. Most well-characterized PLCP functions are associated with germination, senescence, or stress responses. Our study identifies *Glyma.08G116300* as a seed-development-associated PLCP that preferentially accumulates during early-to-middle seed development, with peak expression at the Seed-s5 stage. Heterologous overexpression of *Glyma.08G116300* in *Arabidopsis* increased 1000-seed weight by 12–15% and also increased seed length and width, suggesting that this protease has the capacity to positively influence seed size in a heterologous plant background. This finding expands the potential functional repertoire of PLCPs and raises the possibility that certain PLCP family members contribute to seed-size regulation rather than acting primarily in storage protein degradation after germination.

The developmental expression divergence of *Glyma.08G116300* between CSSL3068 and Nannong 1138-2, higher in CSSL3068 at 28 DAF but higher in Nannong 1138-2 at 35 and 42 DAF, provides additional evidence linking this gene to seed developmental progression. As a chromosome segment substitution line carrying wild-soybean (N24852) introgressions in the Nannong 1138-2 background (Section 2.1), CSSL3068 offers a near-isogenic platform in which allele-dependent expression dynamics can be compared with minimal background noise. Nevertheless, the stage-dependent direction of this expression difference warrants cautious interpretation, and the endogenous function of *Glyma.08G116300* in soybean seed-weight control still requires loss-of-function and stable transgenic validation.

However, the current phenotypic evidence comes exclusively from *Arabidopsis* heterologous overexpression. We lack loss-of-function knockout and stable soybean overexpression data to confirm its endogenous function in soybean. Therefore, we cannot definitively conclude that *Glyma.08G116300* is an endogenous positive regulator of soybean seed-weight. Future studies should use CRISPR/Cas9 knockout and stable overexpression in soybean, ideally in multiple genetic backgrounds, to determine whether manipulation of *Glyma.08G116300* affects seed size, seed weight, and other yield-related traits. It will also be important to characterize its protease activity, identify its endogenous substrates, and determine whether its physical interaction with GmSW16.1 regulates its stability, activity, or substrate specificity during seed development.

## 4. Materials and Methods

### 4.1. Plant Materials, Yeast Strains, and Vectors

The cultivated soybean (*Glycine max*) cultivar Nannong 1138-2 and wild soybean (*Glycine soja*) accession N24852 were used for library construction and gene expression analysis. *Nicotiana benthamiana* was used for BiFC transient expression experiments. *Arabidopsis thaliana* ecotype Columbia-0 (*Col-0*) served as the recipient for heterologous overexpression transformation. The chromosome segment substitution line CSSL3068 (genetic background Nannong 1138-2, introgressed chromosomal fragment originating from wild soybean N24852) was obtained from the Soybean Research Institute, Nanjing Agricultural University, and was included for comparative transcript profiling of developing soybean seeds.

Y2HGold was used as the bait host yeast strain, and Y187 was used as the library-host strain. Yeast vectors pGBKT7-DNA-BD, pGADT7-AD, positive-control plasmid pGBKT7-53, prey control pGADT7-T, and negative control pGBKT7-Lam were purchased from Takara Biomedical Technology (Beijing, China). BiFC vectors pSPYNE and pSPYCEKK were generously provided by Professor Junyi Gai’s laboratory, Nanjing Agricultural University. *Agrobacterium tumefaciens* strain GV3101 was employed for tobacco transient agroinfiltration and *Arabidopsis* floral dip transformation.

### 4.2. Construction of the Y2H cDNA Library from Developing Soybean Seeds

Fresh soybean seeds of Nannong 1138-2 were harvested at 14, 21, 28, 35, and 42 days after flower (DAF), immediately frozen in liquid nitrogen, and ground into fine powder. Total RNA was extracted using the TRIzol reagent method [31] and further purified with spin column-based cleanup kits. RNA integrity was evaluated via 1.5% (*w*/*v*) agarose gel electrophoresis (Figure 1A). RNA concentration and purity metrics (A260/A280, A260/A230) were quantified using a NanoDrop 2000 spectrophotometer (Thermo Fisher Scientific, Waltham, MA, USA). Equal-mass RNA aliquots from each of the five seed-development stages were combined to yield a total RNA input of 1.2 μg (0.24 μg RNA for each individual developmental stage). First-strand cDNA was synthesized using CDS III primers; the SMART III adaptor was incorporated through reverse-transcriptase terminal transferase activity. Double-stranded cDNA was amplified by long-distance PCR (LD-PCR). LD-PCR cycling profile: initial denaturation at 95 °C for 1 min; 24 cycles consisting of 95 °C for 15 s, 68 °C for 6 min; final extension step 68 °C for 5 min. Amplified ds-cDNA was purified using CHROMA SPIN TE-400 columns to remove short DNA fragments smaller than 300 bp [32].

Purified ds-cDNA was co-transformed together with the linearized pGADT7-Rec vector into competent Y187 yeast cells. Transformed cells were spread onto SD/-Leu agar plates and incubated at 30 °C for 3–5 days. Library titer was calculated according to the formula:

Library titer (cfu/mL) = (Total number of independent colonies)/(Volume of plated cell suspension)(1)

A titer value > 1 × 10^7^ cfu/mL was considered to meet high-quality library criteria. All yeast colonies were collected, resuspended in yeast freezing medium, aliquoted, and stored at −80 °C for subsequent library screening experiments. In addition, the library recombination rate was assessed by PCR amplification of the prey inserts in 48 randomly picked colonies; nearly all screened colonies carried inserts, and the insert sizes were broadly distributed between approximately 500 and 2500 bp, in agreement with the ds-cDNA smear (Figure 1B). The total number of independent recombinant clones was calculated as the library titer multiplied by the total volume of stored aliquot.

### 4.3. Bait-Vector Construction, Autoactivation, and Toxicity Assays

The pGBKT7 vector was linearized via double digestion with restriction endonucleases *Nco*I and *Bam*HI, followed by gel purification of linear vector fragments. Gene-specific primers containing protective bases, restriction enzyme recognition sites, and gene annealing sequences were designed (Supplementary Table S1). Full-length coding sequences (CDSs) of *GmSW16.1* and *GsSW16.1* were amplified by PCR. PCR thermal profile: 94 °C for 3 min; 32 cycles: 94 °C for 30 s, 58 °C for 30 s, 72 °C for 2 min; and final extension at 72 °C for 5 min. PCR amplicons were ligated into the intermediate entry vector pCR8 using T4 DNA ligase and transformed into *Escherichia coli* DH5α competent cells. Positive clones were Sanger-sequenced for sequence verification. Sequence validated plasmids were double-digested with *Nco*I/*Bam*HI; target inserts were ligated into linearized pGBKT7 to generate bait constructs pGBKT7-*GmSW16.1* (BD-GmSW16.1) and pGBKT7-*GsSW16.1* (BD-GsSW16.1). Recombinant bait plasmids were purified for downstream experiments.

Recombinant bait plasmids were transformed into competent Y2HGold yeast. Transformed yeast cells were serially diluted and plated onto SD/-Trp (SDO), SD/-Trp/X-α-Gal (SDO/X), and SD/-Trp/X-α-Gal/AbA (SDO/X/A) agar plates and incubated at 30 °C for 3–5 days. Valid bait constructs produce white colonies on SDO and SDO/X plates and exhibit no colony growth on SDO/X/A plates. The co-transformation combination pGBKT7-53 + pGADT7-T served as positive control; pGBKT7-Lam + pGADT7-T served as negative control. For toxicity testing, yeast strains harboring empty pGBKT7, BD-GmSW16.1, or BD-GsSW16.1 were plated on SD/-Trp medium. Colony size and proliferation rates were compared. Only bait constructs passing both autoactivation and toxicity assays were deployed for library screening.

### 4.4. Yeast Two-Hybrid Library Screening and Point-to-Point Interaction Assays

Y2H library screening was performed following the GAL4-based yeast mating protocol described by Fields and Song [33]. A total of 1 mL frozen glycerol stock of Y187 library cells was combined with Y2HGold bait culture (BD-GmSW16.1). An equal volume of 2 × YPDA liquid medium was added, and co-cultivation proceeded at 30 °C with gentle shaking (30–50 rpm) overnight to accomplish yeast mating.

Mating efficiency (%) = (Number of diploid colonies recovered on SD/Trp/Leu plates)/(Total input haploid bait and library cell count) ×100%(2)

After mating, yeast cells were collected by centrifugation. An aliquot of cell suspension was serially diluted and spread onto DDO (SD/-Trp/-Leu) plates to count total independent diploid clones. The remaining cell suspension was plated onto low-stringency DDO/X/A plates for primary screening. After incubation for 3–5 days at 30 °C, blue positive colonies were picked and re-streaked onto high-stringency QDO/X/A (SD/-Trp/-Leu/-His/-Ade + X-α-Gal + AbA) plates for secondary screening.

Positive yeast isolates surviving high-stringency selection were subjected to colony PCR using universal primers matching the pGADT7-Rec vector backbone. PCR products were separated by agarose gel electrophoresis for preliminary identification of redundant clones with identical insert sizes. Plasmids were isolated from candidate yeast strains and Sanger-sequenced. Insert sequences were annotated by BLAST (National Center for Biotechnology Information) searches against soybean reference genome assembly *Glycine max_Wm82_a4_v1*. Sequences lacking genome matches, carrying erroneous open reading frames (ORFs), or representing redundant clones were discarded. Unique candidate genes were retained for further validation.

Full-length CDSs of prioritized candidate genes were amplified and sub-cloned into the pGADT7-AD prey vector via *Nco*I/*Bam*HI restriction digestion and ligation. Each AD-candidate construct was individually co-transformed with BD-GmSW16.1, BD-GsSW16.1, or empty-BD negative-control plasmid into Y2HGold competent yeast. Transformed yeast suspensions were subjected to 10-fold serial dilution (10^−1^–10^−5^), spotted onto DDO/X/A and QDO/X/A selective plates for point-to-point interaction testing, and incubated at 30 °C for 3–5 days before documentation.

### 4.5. Quantitative α-Galactosidase Activity Assays

Fresh yeast single colonies (2–3 mm diameter) originating from point-to-point co-transformations were inoculated into SD/-Trp/-Leu liquid medium and cultured overnight at 30 °C with shaking until OD_600_ reached 0.5–1.0. α-galactosidase enzymatic activity was assayed using *p*-nitrophenyl-α-D-galactopyranoside as chromogenic substrate. The reaction proceeded at 30 °C; reactions were terminated via addition of stop solution. Absorbance values were measured at OD_410_. α-Gal activity (U/mL) was calculated using the following formula:

α-Gal activity (U/mL) = (A_410_ × Vr × 1000)/(ε × l × Vs × t) (3)

A_410_ = blank-corrected absorbance at 410 nm; Vr = total reaction volume (mL); Vs = volume of yeast-culture supernatant used in assay (mL); t = reaction incubation time (min); ε = molar extinction coefficient of p-nitrophenol (18.5 mmol^−1^·L·cm^−1^); and l = spectrophotometer cuvette light path length (cm). Enzymatic activity values were normalized against the OD_600_ of corresponding yeast liquid cultures. Three independent biological replicates were set for each co-transformation combination. Results are reported as mean ± standard deviation (SD). Statistical significance was evaluated by one-way analysis of variance (ANOVA) followed by Tukey’s honestly significant difference (HSD) post hoc test for multiple comparisons; *p* < 0.05 was considered significant.

### 4.6. Bimolecular Fluorescence Complementation (BiFC) Assays

BiFC acceptor vectors pSPYNE and pSPYCEKK were linearized using restriction endonuclease BamHI. Gene-specific primers containing vector homology arms and restriction site sequences (Supplementary Table S1) were used to amplify full-length CDSs of *GmSW16.1*, *GsSW16.1*, *GmMT4* (*Glyma.18G157800*), and *Glyma.08G116300*. In-fusion homologous recombination was used to generate fusion expression constructs: 35S::GmSW16.1-cYFP, 35S::GsSW16.1-cYFP, 35S::GmMT4-nYFP, and 35S::Glyma.08G116300-nYFP. Recombinant plasmids and empty vector controls were transformed into Agrobacterium tumefaciens GV3101. An Agrobacterium strain harboring p19 viral silencing suppressor was co-infiltrated to boost transient transgene expression [34,35].

Agrobacterium cell pellets were resuspended in infiltration buffer (10 mM MES-KOH pH 5.6, 10 mM MgCl_2_, 150 μM acetosyringone) and adjusted to OD_600_ = 0.6. nYFP fusion carrying bacterial suspension and cYFP fusion carrying suspension were mixed at equal volume ratios. The abaxial leaf epidermis of four-week-old *N. benthamiana* plants was infiltrated using needleless syringes. Negative-control combinations: nYFP + cYFP-GmSW16.1; nYFP + cYFP-GsSW16.1; nYFP-GmMT4 + empty-cYFP; and nYFP-Glyma.08G116300 + empty-cYFP. Post-infiltration tobacco plants were cultivated under a 16 h light/8 h dark photoperiod at 24 °C. At 24 h after agroinfiltration, leaf epidermal cells were imaged with a Zeiss LSM780 laser scanning confocal microscope (Carl Zeiss, Oberkochen, Germany). Imaging parameters: YFP: excitation = 514 nm, emission = 525–560 nm; DAPI nuclear stain: excitation = 405 nm, emission = 420–480 nm. Scale bars are marked within micrograph panels [36].

### 4.7. Arabidopsis Floral Dip Transformation and Transgenic Line Identification

The full-length CDS of *Glyma.08G116300* was recombined into binary overexpression vector pCAMBIA3301 to yield construct pCAMBIA3301-Glyma.08G116300, which was transformed into A. tumefaciens GV3101. Floral dip transformation of Col-0 *Arabidopsis* was performed as described [37]. Infiltration buffer contained 5% (*w*/*v*) sucrose and 0.02% (*v*/*v*) Silwet L-77; Agrobacterium OD_600_ was adjusted to 0.8. T_0_ generation seeds were harvested, dried, and subjected to BASTA (glufosinate-ammonium) selection. Seedlings were sprayed repeatedly with 30 mg/L glufosinate-ammonium aqueous solution. Surviving T_1_ primary transformants were validated via genomic DNA PCR. Independent transgenic lines were propagated by successive self-fertilization to obtain homozygous T_4_ generations. Three independent homozygous overexpression lines (OE-1, OE-7, OE-11) with high transgene expression were selected for qRT-PCR profiling and 1000 seed-weight phenotyping. For 1000-seed weight measurement, wild-type and transgenic plants were grown simultaneously under identical growth-chamber conditions (16 h light/8 h dark, 22 °C, 60% relative humidity). Mature siliques were bulk-harvested separately from at least 3 plants per line, and fully filled seeds were collected, air-dried at room temperature for two weeks, and stored under dry conditions. For each biological replicate, 1000 fully developed seeds were counted manually and weighed using an analytical balance (readability 0.1 mg). Three independent biological replicates were measured per line. The length and width of fifteen seeds per line were measured using a stereomicroscope.

### 4.8. Bioinformatic Analyses

Physicochemical parameters (amino acid length, instability index, aliphatic index, grand average of hydropathicity, GRAVY) for the protein encoded by Glyma.08G116300 were calculated using the ExPASy ProtParam web server [38]. Homology-based three-dimensional protein-structure modeling was performed with SWISS-MODEL [39].

Homologous protein sequences of Glyma.08G116300 were retrieved from multiple representative plant taxa, including *Vigna unguiculata*, *Cicer arietinum*, *Medicago truncatula*, *Arabidopsis thaliana*, *Zea mays*, and additional Brassicaceae and Poaceae species. Multiple sequence alignment was executed using ClustalW integrated within MEGA X software. A neighbor-joining (NJ) phylogenetic tree was constructed with 1000 bootstrap replicates for node support assessment; gaps were treated by pairwise deletion [40]. Public tissue expression FPKM data for *Glyma.08G116300* across soybean organs and seed-development stages were downloaded from Phytozome database (assembly *Glycine max*_Wm82_a4_v1; accessed 12 March 2025).

### 4.9. Quantitative Real-Time PCR (qRT-PCR)

Total RNA were extracted from seeds at each developmental stage as described above (21–42 DAF) and from young leaf tissues of T_4_ homozygous *Glyma.08G116300* overexpression lines and wild-type *Col-0* plants. RNase-free DNase I digestion was performed to eliminate genomic DNA contamination. One microgram of total RNA was used for first-strand cDNA synthesis. qRT-PCR amplifications were carried out using SYBR Green-based real-time PCR reagents. For qPCR analysis of soybean genes, the relative expression levels were quantified using *UBI3* (ribosomal-ubiquitin fusion gene *Glyma.20g141600*) as the internal control [41]. *AtActin2* served as internal reference gene for *Arabidopsis*. Relative transcript abundance was calculated by the 2^−ΔΔCt^ algorithm [42]. Three biological replicates and three technical replicates were included per sample. All qRT-PCR primer sequences are compiled in Supplementary Table S1.

## 5. Conclusions

In summary, we successfully constructed a high-quality Y2H cDNA library representing multiple successive seed-development stages of soybean. Using *GmSW16.1* as bait, 11 candidate interacting proteins were identified. Through multiple orthogonal interaction validation assays, we confirmed that both cultivated-soybean-derived GmSW16.1 and wild-soybean-derived GsSW16.1 physically interact with GmMT4 (Glyma.18G157800) and PLCP-family cysteine protease Glyma.08G116300. A two-glutamine allelic polymorphism within the PolyQ repeat tract does not abolish these protein–protein interactions but significantly modulates their binding strength. Combining our protein interaction data with previously published transcriptional regulation evidence, we propose a testable working hypothesis for a dual-layer regulatory module integrating transcriptional control and direct protein–protein interaction governing the *GmSW16.1*-*GmMT4* regulatory node. *Glyma.08G116300* exhibits preferential expression during soybean seed development. Heterologous overexpression of this gene in *Arabidopsis* significantly increases seed weight; nevertheless, its endogenous physiological function in soybean remains to be verified using soybean transgenic materials. This study delivers new mechanistic insights into seed-weight divergence driven by domestication-relevant allelic variation and provides candidate gene resources for soybean molecular design breeding. Our multi-stage seed library construction workflow also offers a practical methodological reference for tissue-specific interactomics investigations focused on crop seed systems.

## Figures and Tables

**Table 1:** Quality metrics of total RNA extracted from soybean seeds at five developmental stages.

RNA	Concentration (ng/μL)	A260/A280	A260/A230
14 DAF	414.52	2.05	2.11
21 DAF	341.31	2.03	2.18
28 DAF	289.14	2.08	2.09
35 DAF	331.67	2.10	2.10
42 DAF	324.24	2.08	2.06

Note: A260/A280 and A260/A230 ratios indicate nucleic-acid purity; values > 2.0 signify high-quality RNA with minimal contamination.

**Table 2:** Unique candidate GmSW16.1-interacting genes identified by Y2H screening and sequence curation.

Mating Number	Gene	Description	Homologous Gene
T7-16-1	*Glyma.14G156400*	Alcohol dehydrogenase-related	*AT1G77120*
T7-21-1	*Glyma.13G260800*	/	*AT4G30074*
T7-24-1	*Glyma.09G158500*	Bowman–Birk serine protease inhibitor	*/*
T7-26-1	*Glyma.17G039100*	S-Adenosylmethionine synthase	*AT3G17390*
			*AT1G02500*
T7-37-1	*Glyma.09G158700*	Bowman–Birk serine protease inhibitor	*/*
T7-41-1	*Glyma.16G062100*	Ubiquinol–cytochrome c reductase c1	*AT5G40810*
T7-42-1	*Glyma.05G222200*	Nucleotide diphosphatase/pyrophosphatase	*AT1G01050*
			*AT4G29690*
T7-49-1	*Glyma.U020300*	SF2-CUPIN domain-containing protein	*AT2G28490*
			*AT2G24550*
			*AT2G18540*
T7-77-2	*Glyma.16G208900*	Bowman–Birk serine protease inhibitor	*AT1G61420*
T7-82-1	*Glyma.08G116300*	Chymopapain/Papaya proteinase II	*AT1G09850*
			*AT4G11320*
			*AT1G47128*
			*AT3G48340*
			*AT4G35350*
T7-106-2	*Glyma.18G157800*	Plant PEC family metallothionein	*AT2G23240*
			*AT2G42000*

## Data Availability

All data generated or analyzed during this study are contained within this article and Supplementary Materials (Supplementary Table S1: Complete primer sequences used in this study).

## Supplementary Materials

The following supporting information can be downloaded at https://www.mdpi.com/article/10.3390/plants15213414/s1, Table S1: All primer sequences used in this study.

## Abbreviations

Y2H	yeast two-hybrid
BiFC	bimolecular fluorescence complementation
DAF	days after flowering
α-Gal	α-galactosidase
QTLs	quantitative trait loci
ABRE	ABA-responsive element
PolyQ	polyglutamine
PLCP	papain-like cysteine protease
LD-PCR	long-distance PCR
ds-cDNA	double-stranded cDNA
ORF	open reading frame
YFP	yellow fluorescent protein
DAPI	4′,6-diamidino-2-phenylindole
GMQE	global model quality estimate
GRAVY	grand average of hydropathicity
FPKM	fragments per kilobase of transcript per million mapped reads
