# Supplementary material for: Construction of a Yeast Two-Hybrid cDNA Library from Soybean Seeds at Different Developmental Stages and Screening of SW16.1 Interacting Proteins
Source: Plants (Basel). 2026 Nov 3;15(21):3414. doi: 10.3390/plants15213414 (PMC13765546; doi:10.3390/plants15213414)
Supplement: Supplementary file 1 [file plants-15-03414-s001.zip › plants-4608428-supplementary.pdf]

Supplementary Material

Table S1 All primer sequences used in this study.

Primer Name	Primer sequence (5'-3')	Purpose
GmSW16.1-pGBKT7-F	CATGccatggGGATGACACCTTTGCGAGTG	Yeast Two-hybrid
GmSW16.1-pGBKT7-R	CGggatccCTCATGCCTTCCAGCCAAAAC	
Gm16G062100-AD-F	CATGccatggGGATGGCTGGAGGTGTTATTCAG	
Gm16G062100-AD-R	CGggatccCCTAGTTGACAACATCAAGAA	
Gm18G157800-AD-F	CATGccatggGGATGGCTGATACAGGTGG	
Gm18G157800-AD-R	CGggatccCCTAAGTGCGGCAGGAGG	
Gm08G116300-AD-F	CCcatatgATGGGTTTCCTTGTGTTG	
Gm08G116300-AD-R	CCcccgggTCAAAGAGGAGAGTGATCA	
Gm05G222200-AD-F	CATGccatggGGATGGGTTCCAATCCCTTG	
Gm05G222200-AD-R	CGggatccCTTATGCAGCAGATAGTAG	
Gm14G156400-AD-F	CATGccatggGGATGCGTGCAGCTGCGGTG	
Gm14G156400-AD-R	CGggatccCTTAGGCCTTCTCCCACTTG	
pGBKT7 primer	GTAATACGACTCACTATAGGGCGA	Sequence
3'AD primer-R	AGATGGTGCACGATGCACAG	
AD T7 primer-F	TTAATACGACTCACTATAGGGCGA	
SPYC-SW16.1-F	GGCGCGCCACTAGTggatccATGACACCTTTGCGAGT	BiFC
SPYC-SW16.1-R	CAGTACTATCGATggatccTGCCTTCCAGCCAAAACC	
SPYN-Gm08g116300-F	GGCGCGCCACTAGTggatccATGGGTTTCCTTGTGTTG	
SPYN-Gm08g116300-R	CAGTACTATCGATggatccAAGAGGAGAGTGATCA	
SPYN-Gm18g157800-F	GGCGCGCCACTAGTggatccATGGCTGATACAGG	
SPYN- Gm18g157800-R	CAGTACTATCGATggatccAGTGCGGCAGGAGGCA	
Gm08g116300-qpcr-F	AAGCGGTTGGGCGTTTTCTG	qRT-pcr
Gm08g116300-qpcr-R	TTCGCTTTCTTCCACACAGTCT	
UBI3-F	GTGTAATGTTGGATGTGTTCCC	
UBI3-R	ACACAATTGAGTTCAACACAAACCG	
AtActin2-qPCR-F	TTCCAGCAGATGTGGATCTCC	
AtActin2-qPCR-R	TTTAAGCCTTTGATCTTGAG	
35S+ Gm08g116300-F	ctctctctcaagcttggatccATGGGTTTCCTTGTGT	Over-expression
35S+ Gm08g116300-R	ctcctgcagctcgatggatccTCAAAGAGGAGAGTG	
